# Comprehensive 16S rRNA gene sequencing and meta-transcriptomic analyses of the female reproductive tract microbiota: two molecular profiles with different messages

**DOI:** 10.1093/hropen/hoag001

**Published:** 2026-01-06

**Authors:** Alberto Sola-Leyva, Inmaculada Pérez-Prieto, Analuce Canha-Gouveia, Eduardo Salas-Espejo, Nerea M Molina, Eva Vargas, Apostol Apostolov, Amruta D S Pathare, Sergio Vela-Moreno, Susana Ruiz-Durán, Bárbara Romero, Rocío Sánchez, José Antonio Castilla-Alcalá, Merli Saare, Ganesh Acharya, Andres Salumets, Signe Altmäe

**Affiliations:** Celvia CC, Tartu, Estonia; Division of Obstetrics and Gynaecology, Department of Clinical Science, Intervention and Technology, Karolinska Institute, Stockholm, Sweden; Department of Gynaecology and Reproductive Medicine, Karolinska University Hospital, Stockholm, Sweden; Celvia CC, Tartu, Estonia; Department of Biochemistry and Molecular Biology, Faculty of Sciences, University of Granada, Granada, Spain; Department of Biochemistry and Molecular Biology, Faculty of Sciences, University of Granada, Granada, Spain; Instituto de Investigación Biosanitaria ibs.GRANADA, Granada, Spain; Department of Physiology, Faculty of Veterinary Medicine, University of Murcia, Murcia, Spain; Department of Biochemistry and Molecular Biology, Faculty of Sciences, University of Granada, Granada, Spain; Instituto de Investigación Biosanitaria ibs.GRANADA, Granada, Spain; Department of Biochemistry and Molecular Biology, Faculty of Sciences, University of Granada, Granada, Spain; Instituto de Investigación Biosanitaria ibs.GRANADA, Granada, Spain; Department of Biochemistry and Molecular Biology, Faculty of Sciences, University of Granada, Granada, Spain; Systems Biology Unit, Department of Experimental Biology, Faculty of Experimental Sciences, University of Jaen, Jaen, Spain; Celvia CC, Tartu, Estonia; Division of Obstetrics and Gynaecology, Department of Clinical Science, Intervention and Technology, Karolinska Institute, Stockholm, Sweden; Department of Gynaecology and Reproductive Medicine, Karolinska University Hospital, Stockholm, Sweden; Department of Biotechnology, Institute of Molecular and Cell Biology, University of Tartu, Tartu, Estonia; Celvia CC, Tartu, Estonia; Division of Obstetrics and Gynaecology, Department of Clinical Science, Intervention and Technology, Karolinska Institute, Stockholm, Sweden; Department of Gynaecology and Reproductive Medicine, Karolinska University Hospital, Stockholm, Sweden; Celvia CC, Tartu, Estonia; Department of Obstetrics and Gynaecology, Institute of Clinical Medicine, University of Tartu, Tartu, Estonia; Instituto de Investigación Biosanitaria ibs.GRANADA, Granada, Spain; Department of Obstetrics and Gynaecology, Virgen de las Nieves University Hospital, Granada, Spain; Instituto de Investigación Biosanitaria ibs.GRANADA, Granada, Spain; Reproduction Unit, Department of Obstetrics and Gynaecology, Virgen de las Nieves University Hospital, Granada, Spain; Instituto de Investigación Biosanitaria ibs.GRANADA, Granada, Spain; Reproduction Unit, Department of Obstetrics and Gynaecology, Virgen de las Nieves University Hospital, Granada, Spain; Instituto de Investigación Biosanitaria ibs.GRANADA, Granada, Spain; Department of Human Anatomy and Embryology, Faculty of Medicine, University of Granada, Granada, Spain; Celvia CC, Tartu, Estonia; Department of Obstetrics and Gynaecology, Institute of Clinical Medicine, University of Tartu, Tartu, Estonia; Division of Obstetrics and Gynaecology, Department of Clinical Science, Intervention and Technology, Karolinska Institute, Stockholm, Sweden; Center for Fetal Medicine, Karolinska University Hospital, Stockholm, Sweden; Women’s Health and Perinatology Research Group, Department of Clinical Medicine, UiT-The Arctic University of Norway, Tromsø, Norway; Celvia CC, Tartu, Estonia; Division of Obstetrics and Gynaecology, Department of Clinical Science, Intervention and Technology, Karolinska Institute, Stockholm, Sweden; Department of Gynaecology and Reproductive Medicine, Karolinska University Hospital, Stockholm, Sweden; Department of Obstetrics and Gynaecology, Institute of Clinical Medicine, University of Tartu, Tartu, Estonia; Division of Obstetrics and Gynaecology, Department of Clinical Science, Intervention and Technology, Karolinska Institute, Stockholm, Sweden; Department of Gynaecology and Reproductive Medicine, Karolinska University Hospital, Stockholm, Sweden; Department of Biochemistry and Molecular Biology, Faculty of Sciences, University of Granada, Granada, Spain; Instituto de Investigación Biosanitaria ibs.GRANADA, Granada, Spain

**Keywords:** endometrial microbiome, microbiota, 16S rRNA gene, meta-transcriptome, dysbiosis, microbial activity

## Abstract

**STUDY QUESTION:**

Does the analysis of endometrial microbes provide the same information when using DNA or RNA sequencing-based techniques?

**SUMMARY ANSWER:**

DNA vs RNA-based microbial analysis techniques demonstrated significant microbial compositional differences and lack of transcriptionally active lactobacilli in the endometrium.

**WHAT IS KNOWN ALREADY:**

Our understanding of the endometrial microbiome is primarily based on DNA-based 16S rRNA gene profiling, but DNA detection does not imply the presence of living microbes. While this method is cost-effective and widely used, it has notable limitations, including the underestimation of microbial diversity, abundance, and functionality, as well as limited species-level resolution. While the microbiome reflects DNA-based characterization, the microbiota more precisely captures metabolically active communities. In this context, meta-transcriptomic analysis, an RNA-based approach, addresses these shortcomings by capturing functional transcripts that are actively expressed in living microbes.

**STUDY DESIGN, SIZE, DURATION:**

This cross-sectional study consisted of 49 reproductive-aged women (27–42 years old) who were receiving ART. By simultaneously analysing the microbial composition and gene expression within female reproductive tract samples, we sought to provide a more comprehensive understanding of the microbiota and functional potential of these samples.

**PARTICIPANTS/MATERIALS, SETTING, METHODS:**

Vaginal swabs, endometrial brushing, and endometrial biopsy samples were collected from 49 participants during the mid-secretory phase of their menstrual cycle, 6–9 days after the luteinizing hormone surge for parallel 16S rRNA gene sequencing and meta-transcriptome analyses. For DNA-based analysis, the 16S rRNA gene V4 region was sequenced. For RNA-based analysis, total RNA was extracted followed by ribosomal RNA depletion. Strand-specific total RNA sequencing libraries were prepared and sequenced. Taxonomy was assigned by using Kraken2 (v2.2.1), and Bracken (v2.7).

**MAIN RESULTS AND THE ROLE OF CHANCE:**

Our findings suggest that in low-microbial-biomass environments such as the endometrium, the correlation between 16S rRNA gene sequencing and meta-transcriptomics is relatively weak. This highlights the limitations of microbial analysis of low-microbial-biomass samples. Alternatively, microbial functions and genome activity may be tissue-specific and dependent on the host tissue environment. Moreover, RNA-based analysis provides higher resolution in detecting certain pathogens, even within the endometrium.

**LARGE SCALE DATA:**

The data presented in the study are deposited in the NCBI SRA Database, accession number PRJNA1247240.

**LIMITATIONS, REASONS FOR CAUTION:**

High levels of host RNA and the low abundance of microbial reads in the endometrium complicate microbial identification. Our findings indicate that RNA-seq enables precise profiling of the vaginal microbiome and, in cases of dysbiosis, reveals higher pathogen activity than DNA-based approaches. However, the limited sample size restricts the generalization of these conclusions.

**WIDER IMPLICATIONS OF THE FINDINGS:**

Contrary to the general belief of the dominance of *Lactobacillus* in the human endometrium, our study suggests that the endometrial microenvironment may be harbouring DNA fragments and/or cells of lactobacilli originating from the lower reproductive tract. Our study results indicate a need to re-consider/re-analyse the endometrial microbiome in health and disease.

**STUDY FUNDING/COMPETING INTEREST(S):**

This work was supported by the projects Endo-Map PID2021-127280OB-I00, ROSY CNS2022-135999, and ENDORE SAF2017-87526-R funded by MICIU/AEI/10.13039/501100011033 and by FEDER, EU. This work was also supported by the Estonian Research Council grants (PSG1082 and PRG1076), Swedish Research Council grant no. 2024-02530 and Novo Nordisk Foundation grant no. NNF24OC0092384. Additionally, A.S.L. and I.P.P. acknowledge Becas Fundación Ramón Areces para Estudios Postdoctorales—Convocatorias XXXV and XXXVI, para Ampliación de Estudios en el Extranjero en Ciencias de la Vida y de la Materia. A.S. is supported by Horizon Europe (NESTOR, grant no. 101120075) and the Ministry of Education and Research Centres of Excellence grant TK214 name of CoE. All the authors declare no conflict of interest.

WHAT DOES THIS MEAN FOR PATIENTS? Microbes play an important role in reproductive health and are linked to conditions such as bacterial vaginosis, candidiasis, endometritis, infertility treatments with poor outcomes, and pregnancy complications. Traditionally, we have studied these microbes by detecting their DNA, which shows which species are present, but not which ones are active. To understand microbial activity, here we additionally use meta-transcriptomics (sequencing all the RNA in a sample), but this approach is challenging because samples often contain little microbial material and are difficult to preserve. In this study, we show that in the endometrium (the inner lining of the uterus), microbial activity is not dominated by *Lactobacillus*, the most common vaginal microbe, and that RNA-based analysis can reveal a different picture. Importantly, signs of microbial activity are detected mainly in specific cases where potentially harmful microbes are present.

## Introduction

The human microbiota, comprising distinct communities of microorganisms located within specific niches throughout the human body and their associated ‘theatre of activity’, has become a central point of medicine (Joos *et al.*, 2025). Understanding the composition and function of these microbial ecosystems has improved our understanding of the symbiotic relationships between microbes and their hosts. High-throughput sequencing technologies have revolutionised the field of microbiota research, enabling comprehensive analysis of microbial communities in various body locations.

Traditionally, microbial composition has been assessed primarily via DNA-based approaches such as 16S rRNA gene sequencing and shotgun metagenomics. Although widely used, DNA-based sequencing techniques identify only microorganisms and genes in a community (i.e. microbiome) without offering insights into microbe viability or (transcriptional) activity (Knight *et al.*, 2018; Molina *et al.*, 2021). As a complementary approach, assessment of microbial gene expression can be achieved through the implementation of meta-transcriptomics, i.e. analysing the total collection of microbial RNA transcripts in a community, to understand the functional activity, microbial composition (i.e. microbiota), and potential contributions to human physiology and pathophysiology (Pereira-Marques *et al.*, 2024). This approach transcends the static snapshot provided by DNA-based analyses, offering a dynamic perspective on microbial activity. Meta-transcriptomic profiling has advanced our understanding of the genetic composition and transcriptional activity within the vaginal microbiome (Fettweis *et al.*, 2019), revealing that species abundance does not necessarily correlate with transcriptional activity (France *et al.*, 2022). Additionally, meta-transcriptomics enables the prediction of impending shifts in the community composition of microbes (France *et al.*, 2022). However, technical challenges, such as the short half-life of RNA (Deutscher, 2006), the high proportion of host-derived RNA compared with scarce microbial RNA, the abundance of rRNA (Pereira-Marques *et al.*, 2024), and the absence of a poly(A) tail in the mRNAs of most prokaryotes (Altmäe *et al.*, 2020), limit its use in certain experimental setups.

The endometrium, once believed to be a sterile environment, is now recognized as a potential niche for microbial colonization and has been associated with various gynaecological diseases (Benner *et al.*, 2018; Molina *et al.*, 2020). Modulation of the endometrial microbes seems to be a promising clinical approach to alleviate several gynaecological conditions, such as chronic endometritis, endometriosis, and risk for gynaecological cancers (Chen *et al.*, 2017; Molina *et al.*, 2020; Toson *et al.*, 2022). Despite significant efforts to identify the core endometrial microbial composition, several factors, including methodological and individual-specific variations (Altmäe, 2018; Molina *et al.*, 2020) have made this task difficult. To the best of our knowledge, only two studies have attempted to unravel the activity of endometrial microorganisms by studying their RNA content (Chen *et al.*, 2021; Sola-Leyva *et al.*, 2021). Our previous RNA-based study of the microbiota mapping of endometria from healthy women identified over 5000 microbial transcripts (bacteria, viruses, fungi, and archaea) (Sola-Leyva *et al.*, 2021). However, this first attempt to profile the endometrial microbiota via meta-transcriptomics failed to reveal a body-site-relevant microbial composition, mainly due to the low microbial presence. Further research relying on well-designed and controlled studies is needed to uncover the true potential of meta-transcriptomics in revealing the active microbial environment within the uterus.

This study aims to elucidate the endometrial microbiome and microbiota through a dual approach combining 16S rRNA metagenomic (DNA) and meta-transcriptomic (RNA) analyses of samples obtained from the same woman. By simultaneously deciphering the genetic composition and gene expression of samples from the female reproductive tract, we aim to provide a more comprehensive understanding of its microbial composition and its potential functional consequences.

## Materials and methods

### Study population and design

This cross-sectional study was approved by the Ethics Committee of the Junta de Andalucía (CEIM/CEI 0463-M1-18r). All participants provided written informed consent before enrolment.

**Figure 1.**
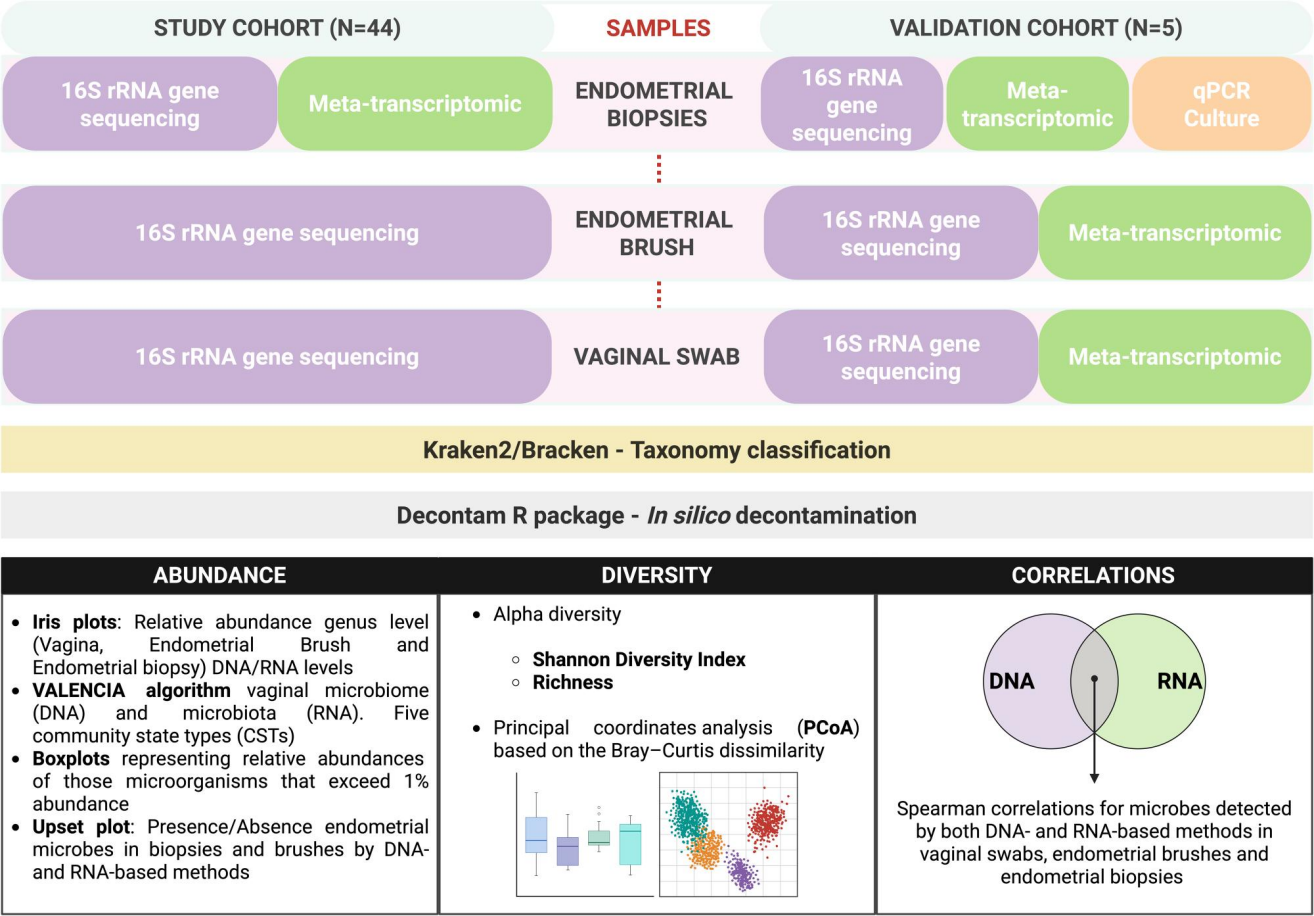
**Study design**. Workflow for characterizing the microbiome (16S rRNA sequencing; DNA-based) and microbiota (meta-transcriptomics; RNA-based) in vaginal swabs, endometrial brushes, and endometrial biopsies. Taxonomy was assigned via the Kraken2/Bracken methodology, and *in silico* decontamination was performed via the Decontam R package. Characterization included abundance, diversity, and correlation between techniques. The study cohort consisted of 44 reproductive-aged women. In the validation cohort (N = 5), a dual approach (DNA- and RNA-based) was applied to all three sample types. Additional validation analyses for endometrial biopsies were performed via real-time quantitative PCR (qPCR) and microbial culture.

The study cohort consisted of 44 reproductive-aged women (27–42 years old) who were receiving ART at the Reproductive Unit at Virgen de las Nieves University Hospital, Granada, Spain, between March 2019 and April 2023 (Fig. 1). Additionally, five women who underwent ART in the same unit were selected for the validation cohort. They provided vaginal, endometrial brush, and endometrial biopsy samples for parallel 16S rRNA gene sequencing and meta-transcriptome analyses. For two women in the validation cohort, endometrial brush samples were not taken. One vaginal swab from the validation cohort could not be analysed. The aim of the validation was to determine whether different microbial detection techniques affect the intra-individual microbial signature, comparing in more detail the DNA- and RNA-based techniques among all sample types (along the reproductive tract), which was not possible for the full cohort (Fig. 1).

The infertility causes of all participants are presented in Supplementary Table S1 and included endometriosis diagnosed by laparotomy or laparoscopy surgery; recurrent implantation failure, defined as repeated implantation failure after the transfer of three good-quality embryos; unexplained infertility; single women undergoing ART; male factor infertility, considering the recommendations of the World Health Organization manual for semen analysis; or tubal obstruction or damage (i.e. tubal factor infertility).

The participants had not received antibiotics within the 3 months preceding sample collection. The exclusion criteria were patients aged ≥43 years, who were receiving hormone therapy, who had gynaecological tumours, systemic diseases, pelvic inflammatory disease, or pelvic pathological conditions other than endometriosis. To synchronize the collection of endometrial samples, all samples were collected at the mid-secretory phase, i.e. 6–9 days post-LH-surge (LH + 6–9), where the ovulation day was estimated with a digital ovulation test (Clearblue, Swiss Precision Diagnostics GmbH, Geneva, Switzerland). The women in the study cohort provided vaginal, endometrial brush, and endometrial biopsy samples for analyses, as described in Fig. 1.

### Sample collection

During the clinic visit, a vaginal swab (eNAT^®^ 606CS01R; COPAN Diagnostics, Brescia, Italy), endometrial brushing sample and endometrial biopsies were collected during the mid-secretory phase of the cycle (LH surge + 6–9 days) by the same gynaecologist. To ensure minimal contamination with bacteria from the lower reproductive tract, an endometrial brush was used with a Tao Brush IUMC (Cook Medical, Madrid, Spain), which was carefully closed within the uterine cavity after sample collection. The samples obtained from the brush were subsequently stored in a Copan eNAT transport system (eNAT^®^ 606C; COPAN) at a temperature of −80°C. During the same procedure, an endometrial biopsy was subsequently taken via a curette device (Gynétics, Medical Products, Hamont-Achel, Belgium). The endometrial biopsies were divided into routine histology evaluation and microbiological analysis. Two additional aliquots were collected and immediately frozen in cryovials via the gas phase of liquid nitrogen for DNA and RNA extraction. These samples were then stored at −80°C until further analysis.

### DNA and RNA isolation

Microbial DNA was extracted from 300 μl of vaginal and endometrial brush samples, which were subsequently resuspended in 2 ml of Copan eNAT transport medium. Approximately 50 ng of endometrial biopsy tissue was used to extract microbial DNA. Microbial DNA extraction was performed with a QIAamp UCP Pathogen Mini Kit (Qiagen, Venlo, Netherlands) and bead beating on a TissueLyser II according to the manufacturer’s instructions, which was automated with a Hamilton STAR robotic platform. DNA quality and purity (230/260/280 nm) were evaluated via a NanoDrop ND-1000 Spectrophotometer (Thermo Fisher Scientific, Waltham, MA, USA), and DNA was quantified via a Qubit 4 fluorometer (Thermo Fisher Scientific).

RNA from vaginal swabs and endometrial brushes was extracted with an RNeasy Plus Universal Mini Kit (Qiagen). A miRNeasy Micro Kit (Qiagen) was used to isolate RNA from up to 30 mg of endometrial tissue, followed by Ribo-Zero plus Kit (Qiagen) processing to remove rRNA. The RNA quality and quantity were checked on a Bioanalyzer TapeStation 4200 (Agilent, Barcelona, Spain) with RNA ScreenTape (Agilent). Additional cleanup via the RNeasy MinElute Cleanup Kit (Qiagen) was performed on RNA from vaginal swabs because the DV200 values were less than 50%. Both the 16S rRNA gene and meta-transcriptomic workflows included extraction and sequencing blanks processed in parallel with biological samples under identical laboratory conditions.

### 16S rRNA gene sequencing

The microbiome was profiled by amplifying the V4 hypervariable region via the primers 515F (5′-GTGYCAGCMGCCGCGGTAA-3′) and 806R (5′-GGACTACNVGGGTWTCTAAT-3′). All PCRs were performed in a 25 μl total volume containing 12.5 μl of 2× KAPA HiFi Hotstart ready mix (Roche Sequencing Solutions (KAPA), Wilmington, MA, USA), 5 μl of each primer (1 mM), and 2.5 μl of undiluted extracted DNA under the following cycling conditions: initial denaturation at 95 °C for 3 min, followed by a cyclic 3-step stage consisting of 35 cycles of denaturation at 95 °C for 30 s, annealing at 55 °C for 30 s, and extension at 72 °C for 5 min. The resulting amplicons were subjected to electrophoresis via 2% agarose gels; the size of each amplicon was approximately 380 bp (base pairs), and DNA quantification was measured via a Qubit 4 fluorometer (Thermo Fisher Scientific). Next, a double purification with magnetic beads was carried out (AMPure XP, Beckman Coulter, Brea, CA, USA). Finally, quality control was evaluated via an HS Bioanalyzer (Agilent) to assess the size of the library and the absence of primer peaks. Illumina Nextera library preparation (Illumina, San Diego, CA, USA) was performed according to the manufacturer’s specifications, combining PhiX phage (20%) with the amplicon library to increase the diversity of the run. The final library was paired-end sequenced (2 × 300 bp) via a MiSeq Reagent Kit v.3 on the Illumina MiSeq sequencing system according to the manufacturer’s instructions.

### Microbiological evaluation by multiplex PCR and bacterial culture

Microbiological analysis of the validation cohort was performed according to the routine workflow of the hospital microbiology laboratory (Sánchez-Ruiz *et al.*, 2025). Briefly, *Lactobacillus* spp., was assessed using a semi-quantitative scale (scant, moderate, or abundant) based on colony counts across successive quadrants of the solid culture plate as previously reported (Sánchez-Ruiz *et al.*, 2025). *Gardnerella vaginalis*, *Enterococcus faecalis*, and a range of pathogens, including *Chlamydia trachomatis*, *Neisseria gonorrhoeae*, *Trichomonas vaginalis*, *Ureaplasma parvum*, *Ureaplasma urealyticum*, *Mycoplasma genitalium*, and *Mycoplasma hominis*, were detected via multiplex PCR on the BD-MAX platform (Becton Dickinson, Sparks, NV, USA). Microorganisms grown in habitual cultures were identified via matrix-assisted laser desorption ionization (MALDI) Biotyper (Bruker Daltonics, Bremen, Germany) or MicroScan (Beckman Coulter) systems.

### RNA sequencing–meta-transcriptomics

The sequencing library was prepared via Illumina Stranded Total RNA Prep, which was subsequently ligated via the Ribo-Zero Plus protocol (Illumina) following the manufacturer’s instructions. The input contained RNA normalized to 1000 ng for endometrial samples and between 500 and 1000 ng total for vaginal samples. Libraries were quantified with a Qubit 4 fluorometer (Thermo Fisher Scientific), and quality control was performed via the fragment analyser TapeStation 4200 (Agilent) with high-sensitivity D1000 ScreenTape (Agilent). RNA sequencing was performed on the NovaSeq 6000 system (Illumina) via an S4 flow cell with paired-end sequencing (100 bp × 2) and a final depth of 60–100 million reads per sample. The RNA sequencing from the validation cohort was conducted on the NextSeq 1000 Illumina platform with paired-end sequencing (50 bp × 2) and a final depth of 60–200 million reads per sample.

### Bioinformatics methods

After 16S rRNA or RNA sequencing, the resulting fastq files were subjected to quality evaluation via FastQC (Babraham Bioinformatics, Cambridge, UK). A per sequence quality score of 30 (Q30) was used, which is a benchmark for quality in next-generation sequencing and highly reduces the probability of error in a read. Reads that successfully passed quality control were processed with the pipeline developed via Nextflow (Di Tommaso *et al.*, 2017). In this pipeline, Kraken2 (Wood *et al.*, 2019) was the aligner used to classify the reads in their corresponding taxa. To perform the taxonomic classification, the standard database (October 2023 version) provided by Kraken2 was used as a reference, setting the minimum hit groups option to 3 and using the paired flag. As a result, a Kraken report file was obtained for every sample, which contained the sample read classification at multiple levels. To estimate the microbe abundance at a single level in the taxonomic tree, the Kraken reports were used as inputs for Bracken (Lu *et al.*, 2017). This analysis used the same database as the Kraken2 process as a reference, with a k-mer length of 50. As a default, the reads were classified to the genus level, and the read threshold was set to 10, meaning that any microbial genus with 10 or fewer reads in the input Kraken report would not receive any additional reads from higher taxonomy levels when the reads were distributed for abundance estimation.

### Microbial decontamination

Given the need for stringent contamination control when characterizing low-microbial-biomass sites such as the upper reproductive tract, we employed an *in silico* decontamination approach via the Decontam R package (v.1.6.0) (Davis *et al.*, 2018; O’Callaghan *et al.*, 2020) in R v4.2.1 (R Core Team, Vienna, Austria) under RStudio v2022.07.2 (Posit Software, Boston, MA, USA). This method identifies contaminating DNA features, allowing us to remove them in downstream analyses. Decontam presents two contamination identification methods: the ‘frequency method’, which is based on the DNA concentration, and the ‘prevalence’ method, in which the presence/absence of a feature in the samples is compared to that in negative controls to identify contaminants (Davis *et al.*, 2018). Recently, Decontam has been validated for meta-transcriptomic analysis by applying a modified frequency method based on the total read counts per sample as material genetic quantity input (Piro and Renard, 2022). We set the Decontam threshold at 0.1 by default to identify contaminating phylotypes. To ensure comparability, the same frequency approach was used to decontaminate the 16S rRNA data.

### Statistical analyses

Statistical analyses were performed via R statistical software v.4.2.1 under RStudio v.2022.07.2. The microbial data generated after taxonomic assignment via both 16S rRNA gene sequencing and meta-transcriptomic data were aggregated to the genus level for diversity and abundance comparisons. The normality of the variables was assessed via the Shapiro–Wilk test. The relative abundances of the identified genera did not conform to a normal distribution and were therefore analysed via the nonparametric Mann–Whitney *U* test. The Benjamini–Hochberg method, the false discovery rate, was utilized to calculate adjusted *P*-values for multiple comparisons. Differences were considered statistically significant between groups when adjusted *P* was <0.05. Alpha diversity indices, including the Shannon diversity index and observed genera richness, were calculated via the ‘diversity’ and ‘specnumber’ functions from the ‘vegan’ package in R. Differences in these diversity indices and abundances among sample groups were assessed via the Mann–Whitney *U* test. Additionally, alpha-diversity comparisons between different sample types from the same woman were conducted via the Wilcoxon signed-rank test for paired data. For beta diversity analysis, Bray–Curtis dissimilarity was computed with the ‘vegdist’ function in R, and permutational analysis of variance (PERMANOVA) was conducted via the ‘adonis’ function from the ‘vegan’ package to evaluate differences in microbial community composition. Furthermore, the vaginal microbiome was classified into five community state types (CSTs) established by the VALENCIA algorithm (France *et al.*, 2020). VALENCIA is a well-established, nearest-centroid-based method that assigns vaginal samples to five CSTs on the basis of their similarity score to reference centroids. Species from the *Lactobacillus*, *Gardnerella*, *Prevotella*, *Atopobium*, and *Sneathia* genera were identified for proper CST assignment. For the validation samples, Spearman correlation tests were performed to assess the association between DNA- and RNA-based relative abundances for each genus. In addition, the relationship between DNA- and RNA-based relative abundances was visualized using scatter plots with linear regression lines.

## Results

### Non-mirroring female reproductive tract microbiome/microbiota composition via 16S metagenomic and meta-transcriptomic analyses

A total of 44 reproductive-aged women (age = 34.8 ± 3.5 years; BMI = 24.2 ± 4.2) were included in the initial study cohort (Supplementary Table S1). After the taxonomic composition across the samples was determined by Kraken2/Bracken, *in silico* decontamination via Decontam revealed two contaminants out of 144 (1.39%) bacterial taxa in the vaginal microbiome, 6 out of 560 (1.07%) in the endometrial brush samples, and 10 out of 237 (4.22%) in the endometrial biopsy samples (Supplementary Table S2).

**Figure 2.**
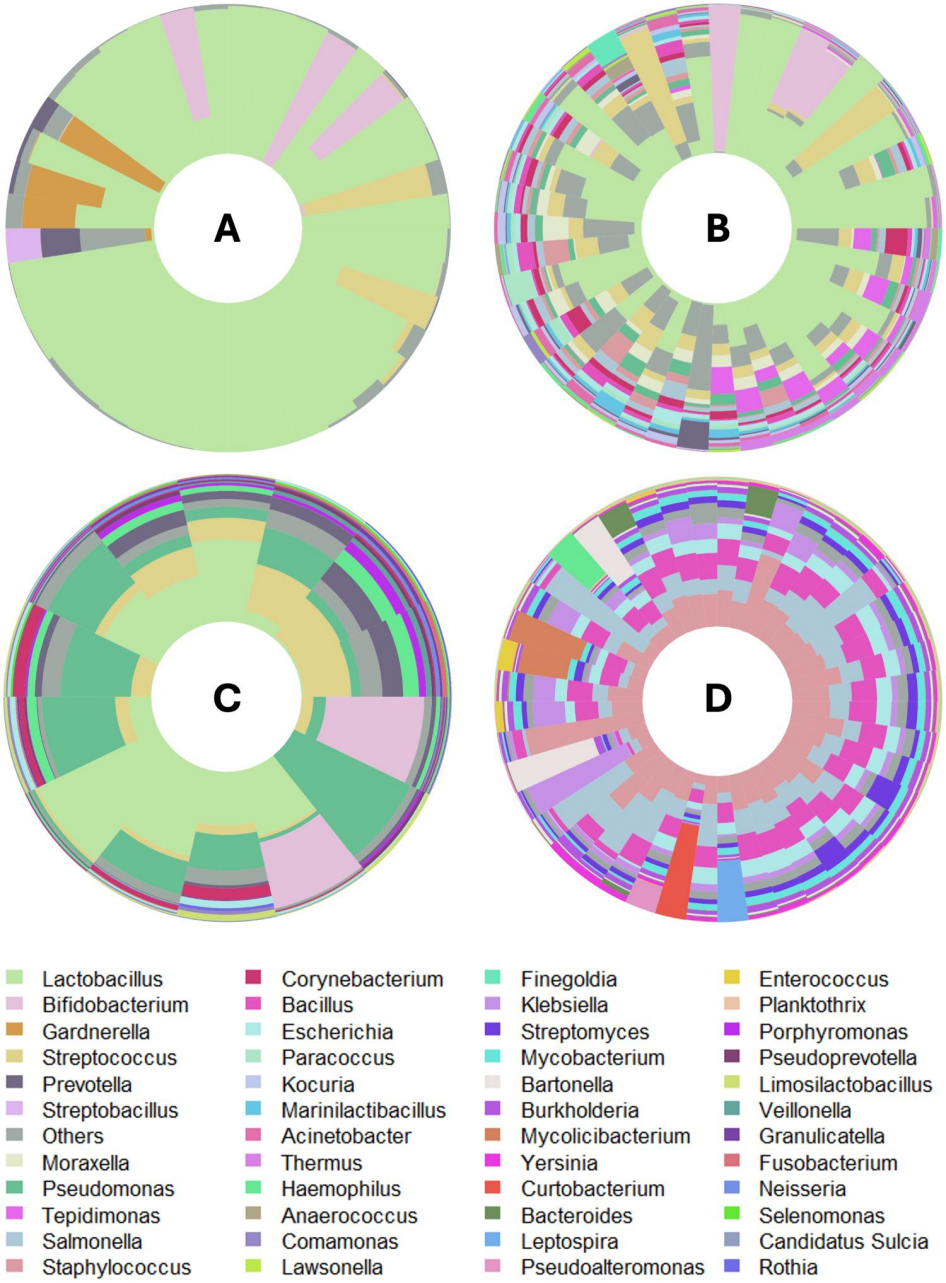
**Microbial composition along the female reproductive tract revealed by DNA- and RNA-based techniques**. Relative abundance of bacterial genera in vaginal (**A**), endometrial brush (**B**), and endometrial (**C**) biopsies detected via 16S rRNA gene sequencing (DNA-based approach). (**D**) Microbial taxa in endometrial biopsies assigned via a meta-transcriptomic (RNA-based) approach. The microbial composition is plotted after decontamination. The samples on the iris plots are arranged by their rotational position around the origin of a principal component analysis (PCA) plot. Centred log ratio (CLR) transformation was applied to normalise the microbial counts at the genus level. Genera with abundances less than 1% were grouped as ‘Others’.

The microbial relative abundances at the genus level for both 16S rRNA gene sequencing and the meta-transcriptome are shown in Fig. 2. Overall, the vaginal microbiome was predominantly composed of *Lactobacillus*, which represents a healthy vaginal microbiome (Fig. 2A). According to the VALENCIA algorithm, 80% of the vaginal samples were classified into *Lactobacillus*-dominant CSTs (i.e. CSTs I, II, III, and V), whereas the remaining 20% had a low abundance of *Lactobacillus* spp. and were assigned to CST IV (Supplementary Table S3). The latter participants exhibited a distinct vaginal microbiome profile, dominated by *Gardnerella*, *Streptococcus*, *Prevotella* or *Bifidobacterium* (Fig. 2A, Supplementary Fig. S1). Similarity scores varied across CSTs, with mean values of 0.39 for CST I, 0.01 for CST II, 0.41 for CST III, 0.59 for CST IV, and 0.05 for CST V. The relatively low scores observed could be explained by the fact that most *Lactobacillus*-dominant samples were composed primarily of *Lactobacillus helveticus* (Supplementary Fig. S1). The endometrial microbiome (DNA-based) was evaluated in the endometrial brush samples (Fig. 2B) and biopsies (Fig. 2C). Compared with those in the vaginal samples, a broader microbial composition, with a marked reduction in the *Lactobacillus* proportion and increases in various other bacterial genera, was detected in the endometrial brush and endometrial biopsy samples via 16S sequencing (Fig. 2A, B, and C).

Different microbes were revealed by analysing the endometrial microbiota via a meta-transcriptomic approach (Fig. 2D). After decontamination, meta-transcriptomic analysis of endometrial biopsies identified 62 out of 523 (11.85%) bacterial taxa as contaminants (Supplementary Table S2). Nonspecific bacterial genera such as *Staphylococcus*, *Salmonella*, *Bacillus*, *Escherichia*, and *Klebsiella* were identified as the most prevalent in the endometrial microbial transcript analysis. This inconsistency may indicate specific active microbial expression, with different genera being transcriptionally active within the endometrial environment or instead reflects poor microbial assignation due to the very low number of bacterial reads compared with human reads.

**Figure 3.**
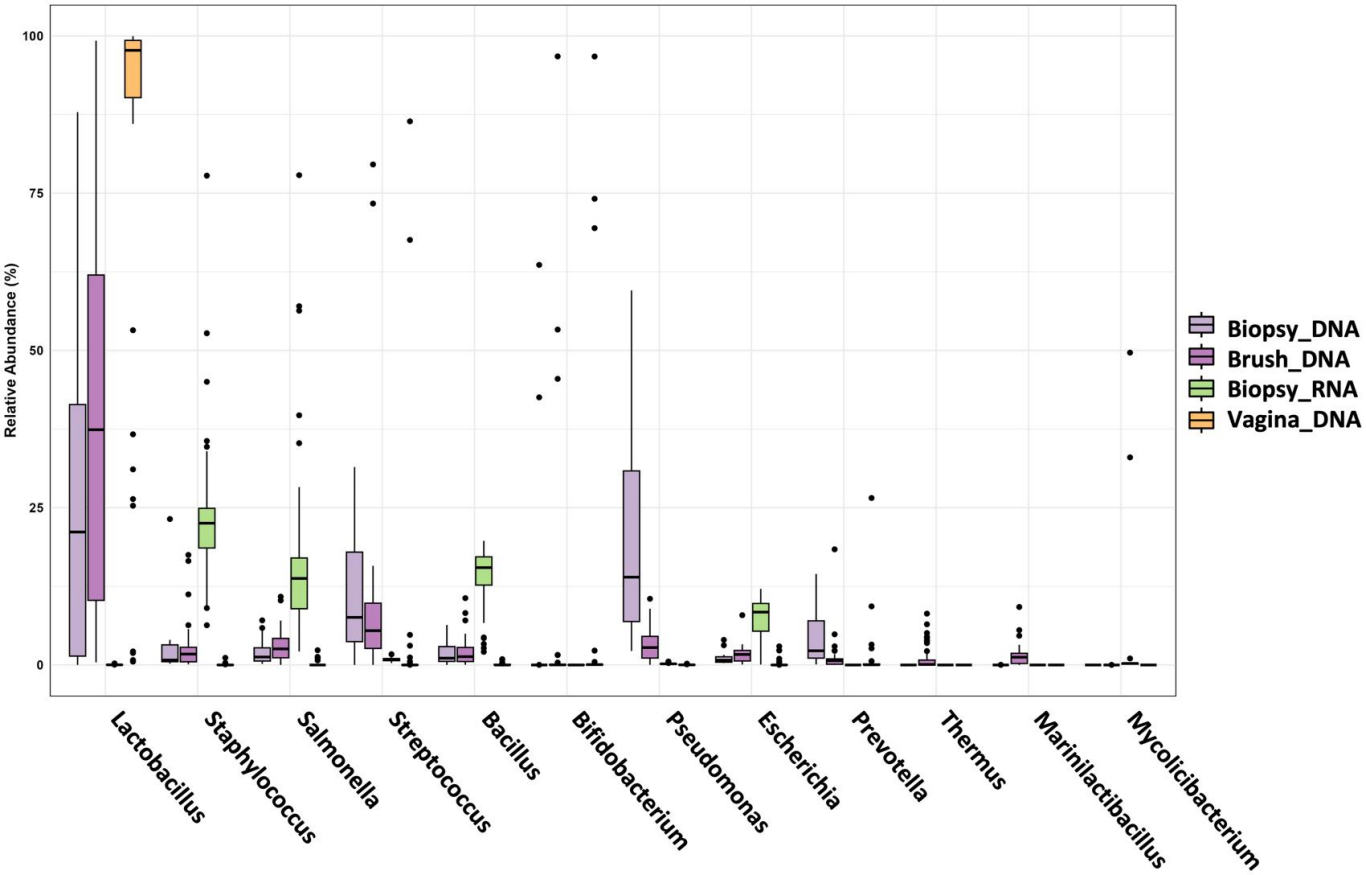
**Boxplots of the most abundant microbes in the vagina and endometrium detected by 16S rRNA gene sequencing and meta-transcriptomic methods**. The boxes represent the interquartile range (IQR) of the data, with the median value indicated by the horizontal line within each box. The whiskers represent the minimum and maximum values, excluding outliers. Sample groups include 16S rRNA gene analysis of vaginal swabs (*Vagina_DNA, orange*), endometrial biopsies (*Biopsy_DNA, light purple*), endometrial brush samples (*Brush_DNA, dark purple*), and meta-transcriptomic data from endometrial biopsies (*Biopsy_RNA, green*). The outliers are shown as individual points. The wider spread and multiple outliers in the endometrial samples indicate substantial variability in microbial abundances across samples.

To illustrate the differences in 16S rRNA gene analysis and meta-transcriptomic approaches for the identification of predominant bacteria in the vagina and endometrium, Fig. 3 displays a boxplot depicting the average relative abundance of those microorganisms that exceeded 1% in abundance and were shared across both anatomical sites. The relative abundance of *Lactobacillus* was greater in samples collected from the vagina than in those collected from the endometrium, as indicated by the median values. Endometrial samples exhibit a wider range of variability, particularly in the meta-transcriptomic analysis of endometrial biopsies (Fig. 3, *Biopsy_RNA* group), which revealed a broad interquartile range (the height of the box) and a significant number of outliers. When comparing the microorganisms identified in endometrial biopsies, a broad range of taxa was observed depending on whether DNA or RNA was analysed. Specifically, lactobacilli were absent in the meta-transcriptome data of endometrial biopsies, indicating low average relative abundance or low genome activity in this tissue environment. On the other hand, other bacteria, such as *Staphylococcus*, *Salmonella*, *Bacillus*, or *Escherichia*, were more frequently detected among RNA transcripts from endometrial biopsies. Additionally, the sample collection method (brush or biopsy) significantly affected the detected microbial community. A comparison of the microbiomes of endometrial brushing and biopsies revealed that the median abundance of lactobacilli was 12.5% greater in the brush group than in the endometrial biopsy group. These results highlight that both the differential analysis of the microbiome/microbiota (DNA/RNA) and the sampling method (brush/biopsy) have a significant effect on the detection of the microbial composition at low-microbial-biomass sites.

### Microbial diversity analyses

**Figure 4.**
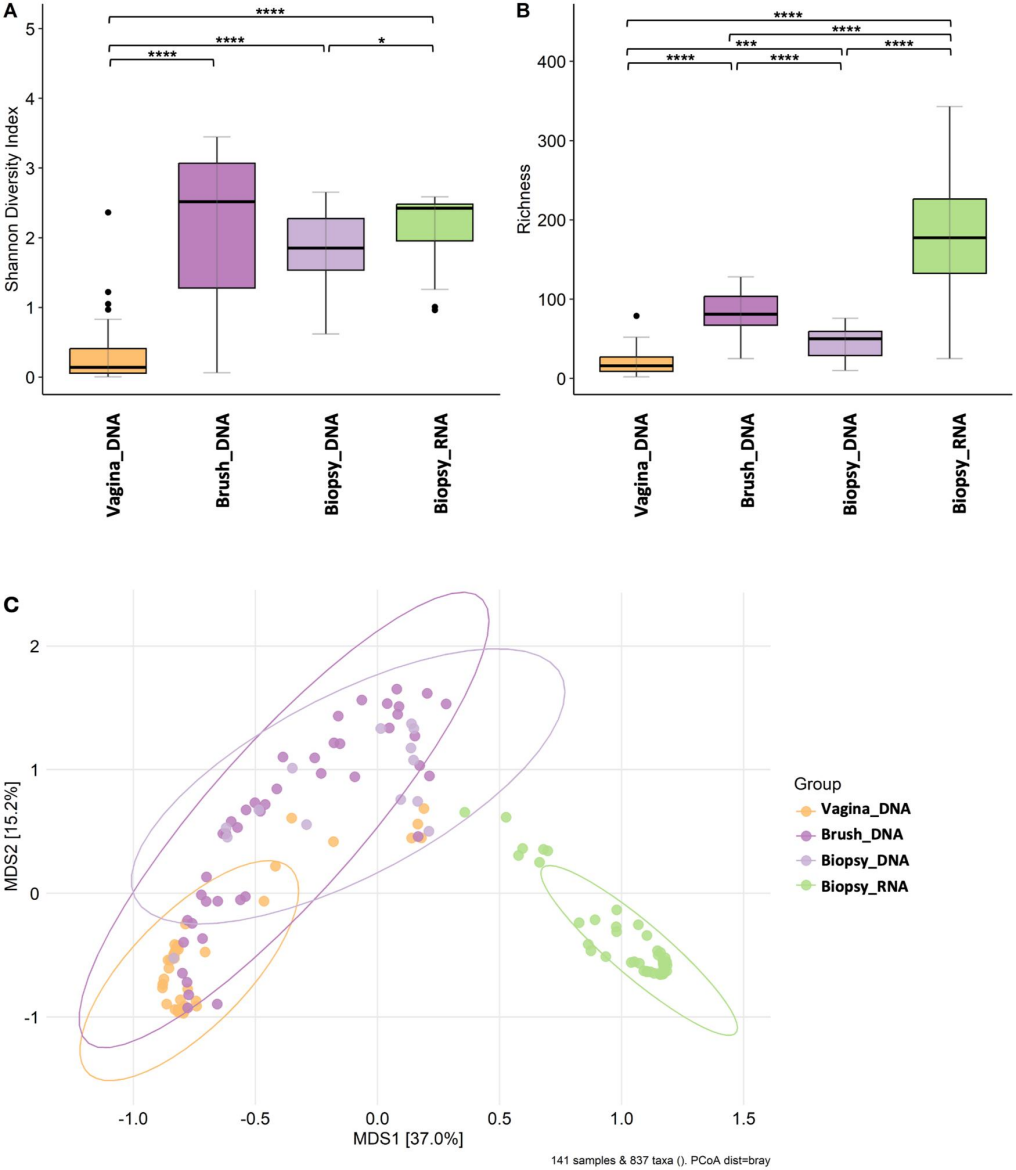
**Microbial alpha and beta diversity measurements in vaginal (DNA), endometrial brush (DNA), and endometrial (DNA and RNA) tissue biopsy samples**. (**A**) Shannon index and (**B**) observed richness. The boxes represent the interquartile range (IQR) of the data, with the median value indicated by the horizontal line within each box. The whiskers represent the minimum and maximum values, excluding outliers. Differences in these diversity indices and abundances among sample groups were assessed via the Mann–Whitney *U* test. Significance levels: ****P < 0.0001; ***P < 0.001; **P < 0.01; *P < 0.05; ns *P* ≥ 0.05. (**C**) Principal coordinate analysis (PCoA) based on Bray–Curtis dissimilarity (Adonis PERMANOVA, all *R*^2^ < 0.05, all *P*-values < 0.001).

Alpha diversity analyses revealed that the Shannon diversity (Fig. 4A) and richness (Fig. 4B) indices (all *P* < 0.0001) of the endometrial microbiome (both brush and biopsy samples) were significantly greater than those of the vaginal microbiome. When different endometrial samples (biopsy/brush) were compared, no statistically significant differences in terms of the Shannon index were found. However, compared with endometrial biopsy, endometrial brushing resulted in greater microbial richness (Fig. 4B; *P* < 0.0001). Additionally, when comparing endometrial biopsy samples analysed via both 16S rRNA gene sequencing and meta-transcriptomic methods, both Shannon diversity and richness were significantly increased according to RNA-seq analysis (all *P* < 0.05) (Fig. 4A and B). Beta diversity analyses of the microbial profile revealed significant dissimilarity between all the groups, i.e. vaginal, endometrial brush, endometrial biopsy-DNA, and endometrial biopsy-RNA (all *P* < 0.001) (Fig. 4C). The distinct clustering indicates that the sampling method (brush vs biopsy) and the type of nucleic acid analysed (DNA vs RNA) influence the detection of the microbial community profile, which emphasises the importance of methodological considerations in microbiome studies.

### Validation revealed the power of meta-transcriptome analysis in microbiota and dysbiosis studies of the lower reproductive tract

**Figure 5.**
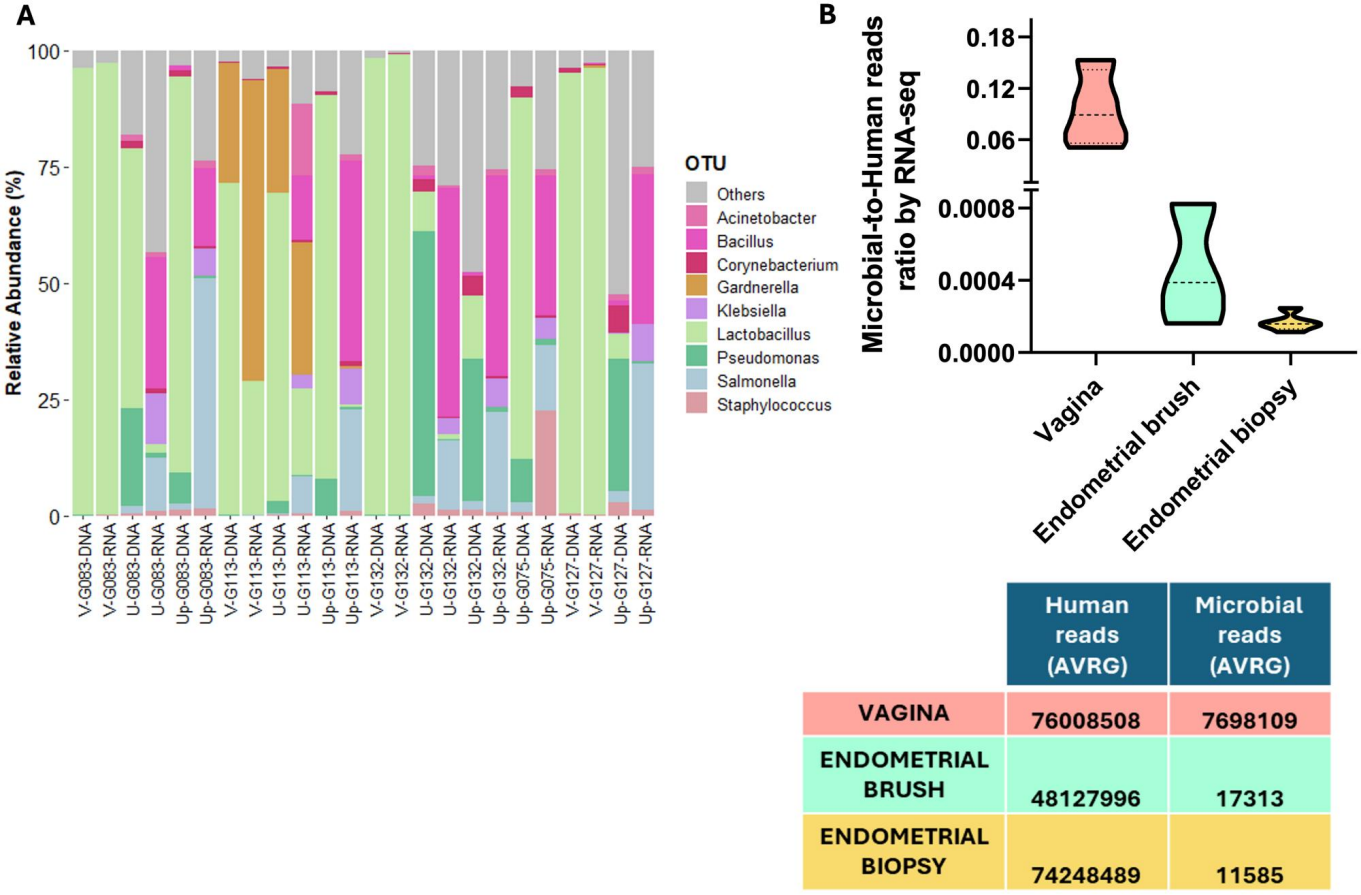
**Microbial composition and read ratios across the validation cohort**. (**A**) Relative abundance of microbial composition (genus level) in the validation cohort from the vagina (V), endometrial brush (U), and endometrial biopsy (Up) samples analysed via 16S rRNA gene sequencing (DNA) and meta-transcriptomic (RNA) methods. (**B**) Ratio of microbial-to-human reads derived from RNA sequencing (RNA-seq) in the vaginal, endometrial brush, and biopsy samples. The data are presented as the median ratio of human to microbial reads within the validation cohort and interquartile range (IQR) of the data. The G075 and G127 participants did not provide endometrial brush samples prior to endometrial biopsy. Vaginal swabs of G075 were not able to be analysed.

We conducted a sub-analysis of the validation cohort in which both DNA- and RNA-based techniques were applied to vaginal swabs, endometrial brush samples, and endometrial biopsies. This validation aimed to determine whether the inconsistencies observed between 16S rRNA gene sequencing (DNA) and meta-transcriptomic (RNA) taxonomic identification were due to sampling (Fig. 4C). To assess whether performing endometrial brushing before biopsy could remove active microbes, contributing to the differences observed between the DNA and RNA profiles, we included a limited number of patients in the validation cohort who underwent direct endometrial biopsies without prior brushing. Notably, even in these cases, *Lactobacillus* remained undetectable at the RNA level (Fig. 5), suggesting that its absence in endometrial biopsies was not an artifact of the sampling sequence but rather a true reflection of its low transcriptional activity in the endometrium.

Figure 5A shows the microbial relative abundances at the genus level in the vaginal (V), endometrial brush (U), and endometrial biopsy (Up) samples, which were analysed via both DNA and RNA sequencing methods within the same samples in the validation cohort. RNA sequencing revealed a significant difference in microbial biomass, i.e. the ratio of microbial-to-human reads (Fig. 5B), with the vaginal swabs having approximately three orders of magnitude more bacterial reads than the endometrial brush and biopsy samples.

Despite differences in microbial identification between 16S rRNA gene sequencing and RNA-seq, *Lactobacillus* was consistently identified as the predominant genus in the vaginal samples, with all samples classified as *Lactobacillus*-dominant CSTs (Supplementary Fig. S2). Notably, vaginal samples analysed via meta-transcriptomic analysis presented a stronger alignment with VALENCIA’s CSTs, which presented higher similarity scores than those obtained via 16S rRNA sequencing (mean scores of 0.73 vs 0.15) (Supplementary Table S3). Specifically, while 16S analysis predominantly identified *Lactobacillus helveticus* as the dominant species in the vaginal samples, RNA-seq revealed *Lactobacillus crispatus* dominance in samples V-G127 and V-G132 (Supplementary Fig. S2). Furthermore, compared with 16S rRNA sequencing (V-G113), RNA-seq provided greater resolution in detecting dysbiotic bacteria, such as *Gardnerella* and *Ureaplasma*, within the vaginal niche (Fig. 5A, Supplementary Fig. S2B). Additionally, the meta-transcriptomic approach, but not 16S rRNA gene sequencing, was the only method capable of detecting *Gardnerella* in endometrial samples. (U-G113, Fig. 5A). These results demonstrate that, compared with 16S rRNA gene sequencing, RNA-level microbial detection provides greater taxonomic resolution and offers a better description of the dysbiotic microenvironment. Furthermore, the endometrial microbiota (based on the RNA profile) and microbiome (based on the DNA detected) were compositionally different. However, when the diversity profiles of the vagina and endometrium were compared via DNA and RNA techniques, no significant differences were detected in the validation cohort, likely because of the small sample size (Supplementary Fig. S3).

**Figure 6.**
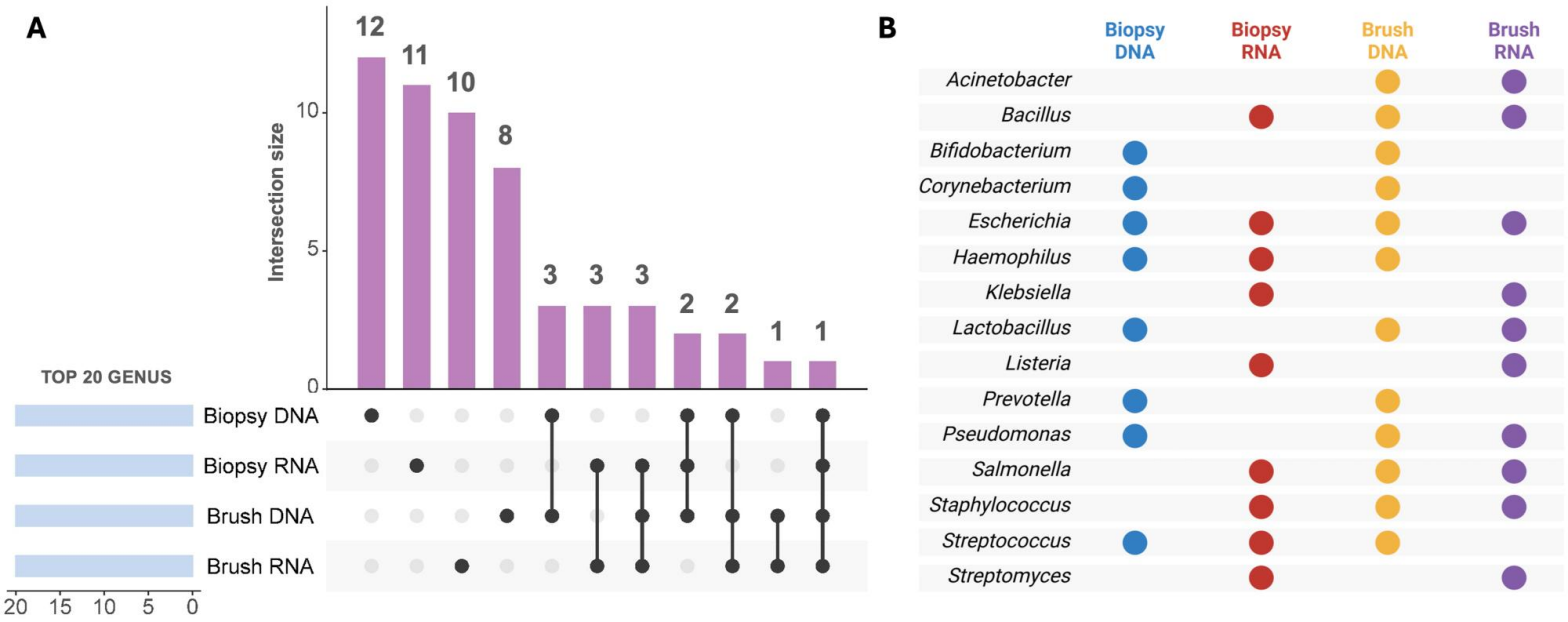
**Concordance analyses between DNA- and RNA-sequencing in endometrium**. (**A**) UpSet plot illustrating the intersection of the top 20 bacterial genera (based on average abundance) detected in endometrial brush and endometrial tissue biopsy samples via 16S rRNA gene sequencing (DNA-based) and meta-transcriptomics (RNA-based). The bar plot represents the intersection size, indicating the numbers of unique and shared genera across different detection methods. The dots in the matrix represent the groups included in the intersection. (**B**) Presence–absence matrix displaying the bacterial genera identified by each method, with dots indicating detection in biopsy DNA (blue), biopsy RNA (red), brush DNA (yellow), and brush RNA (purple).

The presence and absence analysis of the top 20 most abundant microbes in the endometrium across different methodologies (16S rRNA gene sequencing and meta-transcriptomics) and sample types (endometrial brush and biopsy samples) revealed a greater number of uniquely identified microbes than those detected across multiple conditions (Fig. 6A). Despite this, up to 15 shared microbial intersections were observed. Notably, *Lactobacillus* was consistently identified among the top 20 genera under all conditions, except for the meta-transcriptomic analysis of endometrial biopsies (Fig. 6B). *Escherichia* was revealed across all methodologies and sample types. Interestingly, *Klebsiella* and *Listeria* were exclusively detected by RNA-based analysis in both brush and biopsy samples, suggesting potential transcriptional activity despite their lower DNA-based abundance. In contrast, *Bifidobacterium*, *Corynebacterium*, and *Prevotella* were identified only at the DNA level, indicating their genomic presence without any transcriptional signals (Fig. 6B).

**Figure 7.**
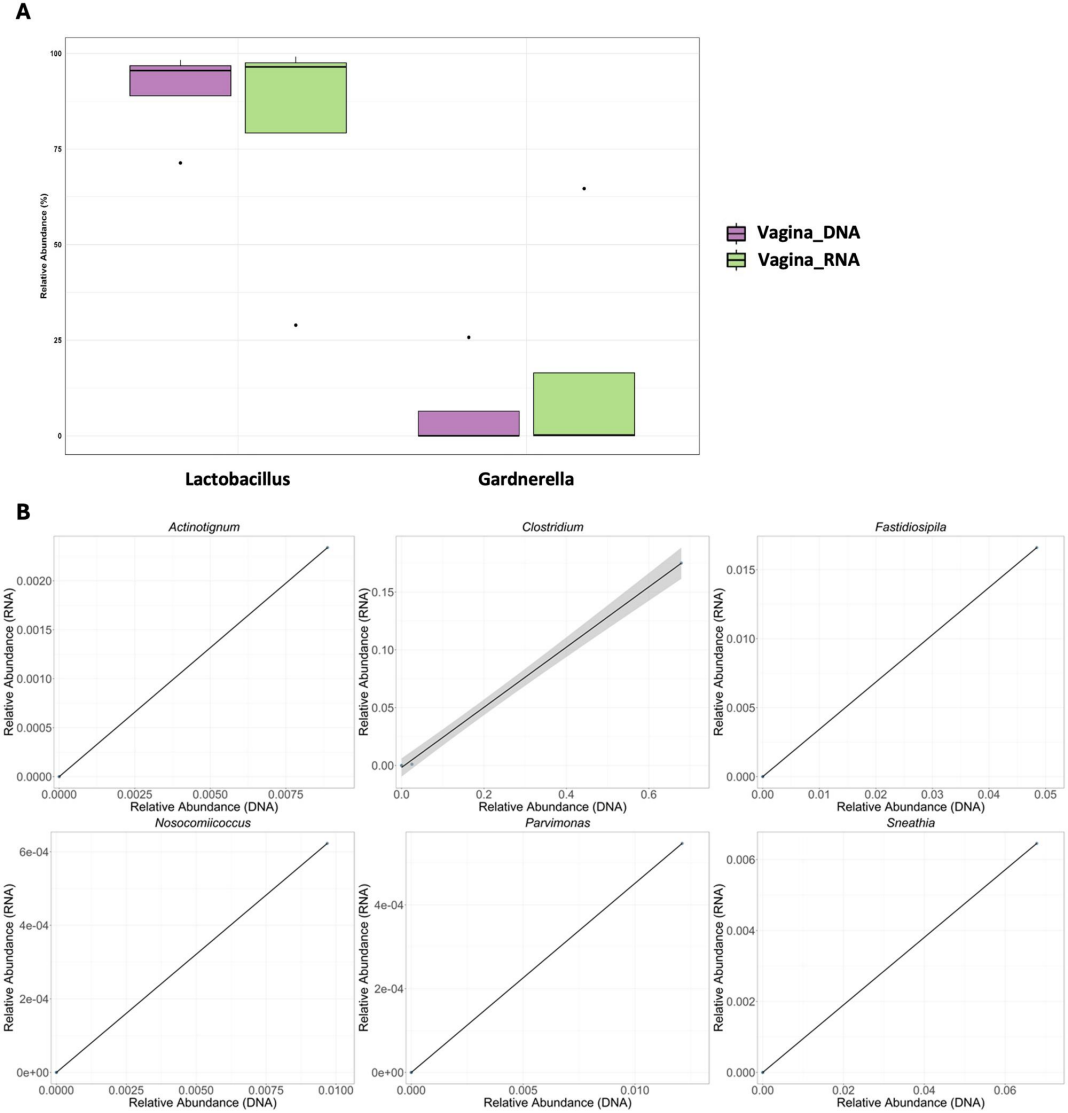
**Correlation analysis of vaginal microbiome and microbiota**. (**A**) Boxplots of the relative abundance of the most abundant microbes in vaginal samples revealed by 16S rRNA gene sequencing (DNA, purple) and meta-transcriptome (RNA, green) analysis. The boxes represent the interquartile range (IQR) of the data, with the median value indicated by the horizontal line within each box. The whiskers represent the minimum and maximum values, excluding outliers. (**B**) Scatter plots showing the relationship between the taxonomic profiles of 16S rRNA gene sequencing (*X* axis) and meta-transcriptomics (*Y* axis). Points represent individual samples. Solid lines indicate linear regression fits, and shaded areas represent the 95% confidence intervals.

The most abundant microbes in the vagina, with a relatively high relative abundance of 1%, were *Lactobacillus* and *Gardnerella* (Fig. 7A). A comparison of the median relative abundances of *Lactobacillus* and *Gardnerella* detected via DNA- and RNA-based techniques revealed no statistically significant differences (Supplementary Table S4A). A total of 45 microbes were identified in the vagina via both 16S rRNA gene sequencing (DNA-based) and meta-transcriptomic (RNA-based) methods. Correlation analysis of microbes detected by DNA and RNA sequencing revealed that 6 out of the 45 dually detected species exhibited a significant positive correlation (all adjusted *P* < 0.05) (Fig. 7B, Supplementary Table S5A). Interestingly, the relative abundances of *Lactobacillus* and *Gardnerella* identified via the DNA and RNA approaches were positively correlated, although the correlations did not reach statistical significance. These findings indicate that these microorganisms were consistently detected across DNA- and RNA-based methodologies, suggesting strong agreement between the two techniques in identifying and quantifying vaginal microbial populations.

**Figure 8.**
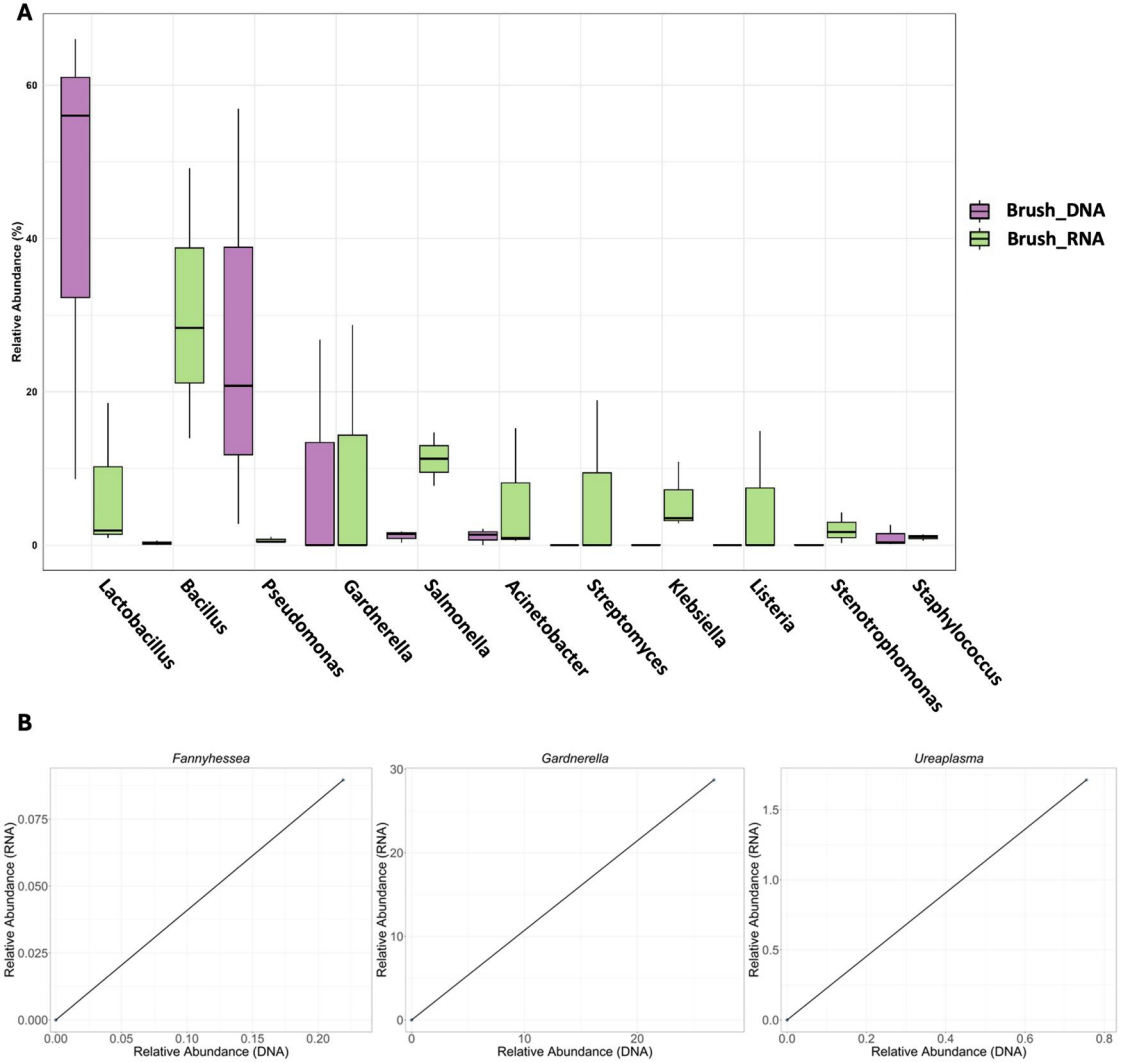
**Correlation analysis of endometrial brush samples microbiome and microbiota**. (**A**) Boxplots of the relative abundance of the most abundant microbes in the endometrial brush samples revealed by 16S rRNA gene sequencing (DNA, purple) and the meta-transcriptome (RNA, green). The boxes represent the interquartile range (IQR) of the data, with the median value indicated by the horizontal line within each box. The whiskers represent the minimum and maximum values, excluding outliers. (**B**) Scatter plots showing the relationship between the taxonomic profiles of 16S rRNA gene sequencing (*X* axis) and meta-transcriptomics (*Y* axis). Points represent individual samples. Solid lines indicate linear regression fits.

For the endometrial brush and biopsy samples, the microbial landscapes revealed through analysis of the 16S rRNA gene and meta-transcriptomic profiling were notably different (Figs 2 and 5A). Specifically, the median relative abundances of the most prevalent (more than 1% relative abundance) and commonly identified taxa in brush samples differed significantly between the two techniques (DNA and RNA), highlighting a lack of consistency in their quantification (Fig. 8A). Focusing on the abundance of lactobacilli, owing to their high relevance in the field, we found a lack of consistency in terms of abundance depending on the detection method used (Fig. 8A). Despite the substantial differences, no statistically significant variation was found in the median abundance detected by DNA- vs RNA-based techniques in the endometrial brush samples, likely due to the limited sample size (Supplementary Table S4B).

Endometrial brush samples analysed by 16S gene/DNA sequencing and meta-transcriptomics/RNA indicated that 24 microbial taxa were present in both analyses. Among them, three bacteria were significantly positively correlated. Specifically, *Fannyhessea*, *Gardnerella*, and *Ureaplasma* were significantly correlated according to both the DNA and RNA detection methods (Fig. 8B, Supplementary Table S5B). A specific qPCR assay performed within the validation cohort to detect pathogens revealed that participant G113 tested positive for *Ureaplasma* together with the development of *Gardnerella vaginalis* and *Lactobacillus jensenii* in culture (Supplementary Table S1). G113 was the only participant in the meta-transcriptomic analysis of the endometrial brush, which revealed a relative abundance of *Lactobacillus* greater than 10% (Fig. 5A) (specifically, 18% *Lactobacillus jensenii*). These findings support the results obtained via DNA and RNA sequencing methods.

**Figure 9.**
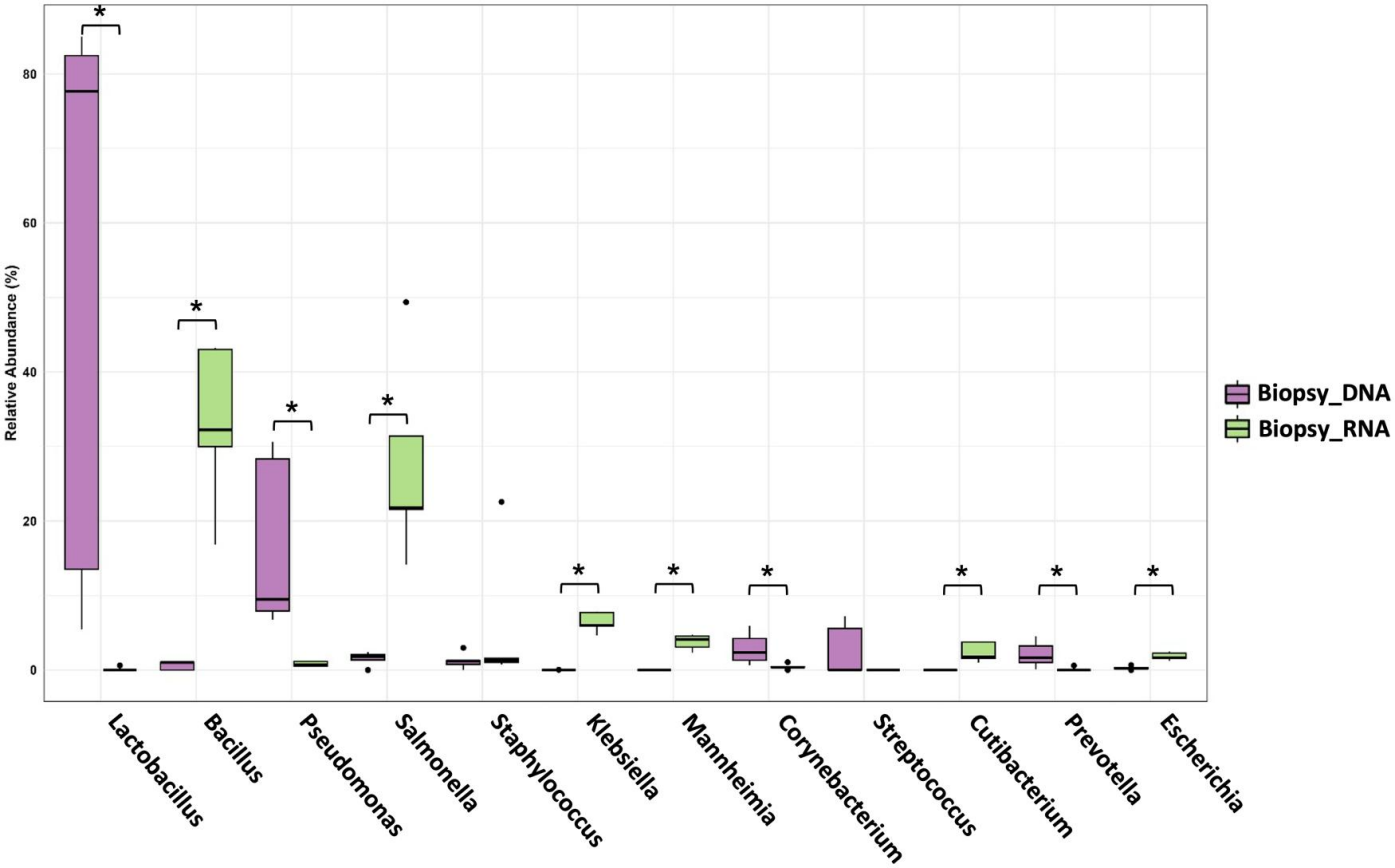
**Analysis of endometrial biopsy samples microbiome and microbiota**. Boxplots of the relative abundance of the most abundant microbes in endometrial biopsy samples revealed by 16S rRNA gene sequencing (DNA, purple) and the meta-transcriptome (RNA, green). Significance was considered for adjusted *P*-values < 0.05 (*), revealed by Mann–Whitney *U* test. The boxes represent the interquartile range (IQR) of the data, with the median value indicated by the horizontal line within each box. The whiskers represent the minimum and maximum values, excluding outliers.

The analysis of microbes in endometrial biopsies revealed the absence of *Lactobacillus* in the meta-transcriptomic data and statistically significant differences in the relative abundances of individual microbes detected by 16S rRNA gene sequencing and meta-transcriptomics. However, the abundances of *Streptococcus* and *Staphylococcus* were similar across both methods (Fig. 9A, Supplementary Table S4C). The positive correlations observed between pathogens detected via DNA- and RNA-based methods in the endometrial brush samples (Fig. 8B) were not consistently significant in the biopsy samples. Among the 18 microbes detected by both DNA and RNA techniques, only one, *Tepidimonas*, remained significant (Supplementary Table S5C).

## Discussion

The endometrial microbial composition has garnered considerable attention within the field of reproductive medicine because of its potential role as a biomarker for both diagnosis and prognosis. Despite the limitations of current methodologies, characterising microbial communities offers promising insights into reproductive health. A key focus in uterine microbiome research has been the detection of *Lactobacillus* species, whose dominance (>90%) has been linked to higher pregnancy rates (Moreno *et al.*, 2022). However, it remains unclear whether the microbiome, defined by bacterial DNA sequences identified in metagenomic studies, corresponds to live microbes. The first comprehensive map of the active endometrial microbiota, defined as the community of living microorganisms inhabiting the endometrium, based on microbial RNA transcripts, revealed cyclic shifts in community composition across the menstrual cycle and potential host–microbiota interactions influencing endometrial receptivity (Sola-Leyva *et al.*, 2021). However, the absence of niche-specific microbes in meta-transcriptomic studies, combined with the lack of multi-omic approaches, makes defining an active and functional endometrial microbiota challenging. Therefore, new studies are needed to clarify whether microbes are present in the endometrium and, if so, which microbes constitute the endometrial microbiota/microbiome. This study is the first to establish a continuous microbial profile from the vagina to the endometrium via a dual approach: DNA-based 16S rRNA gene sequencing and RNA-based meta-transcriptomics, which were validated by qPCR and bacterial culture within the same cohort and samples.

16S rRNA gene sequencing of the female reproductive tract revealed a distinct microbiome in the vaginal, endometrial brush, and endometrial biopsy samples. The method of endometrial sampling significantly impacts microbiome findings (Lüll and Org, 2023). The Tao Brush IUMC Endometrial Sampler is an endometrial sampling device featuring an outer sheath that minimizes lower genital tract contamination. Previous findings using this device identified an endometrial microbiome predominantly dominated by *Bacteroides* (Verstraelen *et al.*, 2016) and *Lactobacillus* (Diaz-Martínez *et al.*, 2021). Investigating the endometrial microbiome from hysterectomy samples, a major surgical procedure involving removal of the uterus, revealed a distinct microbial signature in endometrial tissue, minimizing vaginocervical contamination (Canha-Gouveia *et al.*, 2023). In contrast to expectations, this signature was characterized by the presence of *Acinetobacter*, *Arthrobacter*, *Coprococcus*, *Methylobacterium*, *Prevotella*, *Roseburia*, *Staphylococcus*, and *Streptococcus* (Canha-Gouveia *et al.*, 2023). In line with these findings, our results revealed that *Acinetobacter* was detected at both the DNA and RNA levels only in samples collected with the Tao Brush (Fig. 6B). Additionally, *Streptococcus* and *Staphylococcus* were transcriptionally active, as they were identified at the RNA level, suggesting biological activity beyond the mere presence of DNA sequences. While transcriptional activity of *Klebsiella*, *Escherichia*, and *Staphylococcus* was detected in endometrial samples, the biological relevance of these signals remains uncertain. In low-biomass environments such as the endometrium, distinguishing true microbial activity from environmental or reagent-derived background is particularly challenging. The presence of bacterial RNA suggests metabolic activity but not necessarily persistent colonization or pathogenic processes. Interestingly, similar taxa have been reported in the endometrial microbiome of infertile patients, including associations of *Klebsiella* and *Staphylococcus* with poorer reproductive outcomes (Moreno *et al.*, 2022). Moreover, analogous observations in semen microbiome studies show that multiple genera commonly considered commensal may in fact represent transient colonisers or commensals in reproductive fluids (Davies *et al.*, 2023). Such recurrence across independent reproductive tract niches supports the view that transient microbial trafficking or metabolic by-products may explain these findings, rather than established infection. Functionally, even short-lived microbial presence may modulate the local immune micro-environment through microbial-associated molecular patterns or secreted metabolites. This could in theory influence endometrial receptivity via immune signalling pathways (Bardos *et al.*, 2019). Consequently, these results should be interpreted with caution, acknowledging both the technical limitations of meta-transcriptomic profiling in low-biomass settings and the emerging possibility that incidental microbial signals can still hold biological significance.

In parallel, the use of *in silico* decontamination approaches such as the Decontam R package (Davis *et al.*, 2018) could generate meaningful results by avoiding/reducing microbial contamination. In the studied cohort, more contaminants were identified in endometrial biopsies (4.2% of the identified bacteria) than in vaginal or endometrial brush samples (∼1%). These findings underscore the necessity of integrating sampling and data analysis strategies when conducting microbiome studies in areas with potentially low microbial biomass, such as the endometrium, to minimise the influence of contaminants. Our results highlight the importance of using a sampling method that reduces the risk of contamination from the lower female reproductive tract when screening the endometrial microbial composition.

With respect to the microbiome (DNA-based) and microbiota (RNA-based) of the endometrial samples, our results consistently revealed the presence of *Lactobacillus*, although with significant differences in relative abundance (Figs 8A and 9A). Meta-transcriptomic analysis revealed a lower representation, with *Lactobacillus* being undetectable in biopsies and present at low abundance (∼1%) in some endometrial brush samples (Figs 2D and 6B). These findings align with our previous study, where the abundance of *Lactobacillus* species remained less than 1% in endometrial biopsies (Sola-Leyva *et al.*, 2021). Additionally, our prior research revealed a high presence of *Klebsiella* transcripts, a result corroborated in this study, where *Klebsiella* was detected exclusively by RNA-based techniques in both endometrial brush and biopsy samples (Fig. 6B). This bacterium has also been previously identified in the endometrium via qPCR (Moreno *et al.*, 2018) and 16S rRNA gene sequencing (Moreno *et al.*, 2022; Canha-Gouveia *et al.*, 2023; Zou *et al.*, 2023). This observation may reflect the effect of the brushing procedure in dislodging or redistributing microbial populations within the endometrium, thereby reducing their detectability in subsequent biopsies. Mechanical disturbance during sampling could transiently alter microbial abundance or spatial distribution. Despite this, some enrolled patients did not undergo endometrial brushing before the biopsy, yet they still showed no significant presence of lactobacilli (Fig. 5A).

Transcriptome analysis could not confirm the presence of a ‘core’ microbiome, as identified in DNA studies. These findings suggest that many of the microbes detected at the DNA level may not be part of the normal endometrial microbiome and may not be transcriptionally active. A key finding of this study is the lack of evidence for transcriptionally active *Lactobacillus* in the endometrium, suggesting that these bacteria may reach the uterine cavity but are not metabolically active under the examined conditions. Detection at the DNA level could therefore derive from residual bacterial DNA or non-viable or transcriptionally inactive *Lactobacillus* cells rather than from true active colonisation. However, this result should be interpreted with caution, as the high proportion of host RNA and the low microbial biomass in endometrial samples may limit the detection of low-abundance transcripts. The presence of lactobacilli in the human endometrium and their biological role have been the subject of research for many years (Altmäe, 2023), with questions raised about their ability to survive and thrive in the uterine environment, which does not provide the low pH they typically require (Parratt *et al.*, 1995; Lykke *et al.*, 2021), among other factors. Our results raise doubts about whether lactobacilli are core microbes in the endometrium. Similarly, integrated metagenomic and meta-transcriptomic analyses reveal striking divergences between genetic potential and transcriptional activity, highlighting RNA-seq as indispensable for capturing the true dynamics of microbial communities (Chia *et al.*, 2025). Additionally, the endometrium is not a passive environment; rather, it exhibits tightly regulated immune responses that vary across the menstrual cycle and reproductive states (Bister *et al.*, 2024). Microbes can reciprocally influence immune cell phenotype and function, with immune cells competing for nutrients and mucosal space against potential pathogens. Current evidence suggests that immune cells play a crucial role in maintaining a symbiotic relationship with commensal microbes, but detailed mechanistic understanding is still emerging (Zheng *et al.*, 2020). However, more comprehensive studies are needed to fully elucidate the complex dynamics of immune cell–microbe modulation in the endometrial environment.

The high host RNA background and the low abundance of microbial reads in the endometrium (Fig. 5B) complicate microbial identification (Sola-Leyva *et al.*, 2021; Zhang *et al.*, 2021). Specifically, our results demonstrate how profiling the microbiome via RNA-seq can reveal the vaginal microbiome with high precision. Notably, in cases of dysbiosis, RNA-seq reveals greater pathogen activity than DNA-based techniques do (Supplementary Fig. S2). Furthermore, in eubiotic *Lactobacillus*-dominant microbiomes, the *Lactobacillus* species detected differed between the two methods (Supplementary Figs S1 and S2). Our findings indicate that *Lactobacillus helveticus*, a species absent in the VALENCIA consensus CST database, was detected via DNA-based analyses of the vagina. Given that VALENCIA assigns CSTs on the basis of species-level classification and that 16S rRNA sequencing generally does not achieve species-level resolution, the presence of *L. helveticus* in our 16S rRNA gene analyses of the vaginal microbiome may reflect misclassification, since the RNA from the same samples analysed revealed dominance of *Lactobacillus crispatus*. Comparative genomics studies have demonstrated that *L. helveticus* and *L. crispatus* are closely related (sister) species with significant genomic overlap (Cremonesi *et al.*, 2012; France *et al.*, 2016). This phylogenetic proximity complicates species-level classification via 16S rRNA gene sequencing, as their sequences share a high degree of similarity, making their differentiation unreliable with short-read sequencing approaches.

Profiling of microorganisms in the endometrium remains challenging, as there is no evident correlation between the microorganisms detected in the endometrium via DNA- and RNA-based techniques. Nevertheless, several microbes were associated with a dysbiotic endometrial microenvironment in endometrial brush samples (Fig. 8), such as *Gardnerella* and *Fannyhessea*, which were positively correlated with endometrial brushes, as determined by both DNA and RNA sequencing (Fig. 8B). Our study revealed a greater number of vaginal bacteria whose relative abundances, determined by 16S gene sequencing and RNA transcripts, were strongly correlated (Fig. 7). These findings highlight the diagnostic potential of meta-transcriptomics for characterizing dysbiosis in the female reproductive tract. Meta-transcriptomic profiling has recently been applied to respiratory infections, enabling discrimination of pathogen-specific host responses and offering new avenues for molecular diagnosis (Doxey *et al.*, 2025). In reproductive medicine, transcriptomic analyses are already integral to assessing endometrial receptivity (Meltsov *et al.*, 2023). Meta-transcriptomics may therefore enable a unified approach to simultaneously evaluate both endometrial receptivity and microbial imbalance, offering a comprehensive tool for clinical assessment. In contrast, the number of correlations observed in the endometrial samples was half of that found in the vagina. This disparity suggests that abundance measurements derived from DNA-based methods may not accurately reflect the true microbial activity at low-microbial-biomass sites, potentially leading to a misestimation of the dynamic microbial landscape within the endometrium. Alternatively, the genome activity of specific microbes is dependent on the host condition (Avican *et al.*, 2021), which makes the direct comparison of DNA- and RNA-based profiling approaches even more controversial.

Technically, in samples where host RNA is predominant over microbial RNA, a sizeable portion of sequencing capacity is allocated to the host. As a result, achieving adequate microbial detection requires deep sequencing, which increases both costs and computational demands (Ojala *et al.*, 2023). Consequently, detecting microbial sequences often requires substantially greater sequencing depth and coverage. In this study, we relied on 60–200 million reads per sample. Given that previous studies have employed sequencing depths ranging from 1 (Abu-Ali *et al.*, 2018) to 250 million reads (Ojala *et al.*, 2021), our approach enhances the reliability of microbial detection by ensuring greater sequencing depth. Our previously established pipeline for microbial detection (Sola-Leyva *et al.*, 2021), which uses Kraken2/Bracken, employs a k-mer-based approach. This method has demonstrated greater sensitivity in the taxonomic classification of meta-transcriptomes, particularly in samples with a high human-to-bacteria cell ratio (Ye *et al.*, 2019; Meyer *et al.*, 2022).

## Conclusions

The novelty of this study lies in its parallel dual analysis using DNA- and RNA-based techniques along the female reproductive tract to characterize the microbiome and microbiota. This analysis revealed that in low-microbial-biomass environments such as the endometrium, the correlation between the two techniques is relatively weak, highlighting how the detected microbial landscape varies depending on the chosen approach. Nevertheless, RNA-based analysis provides increased resolution for detecting certain pathogens, even in the endometrium. Our analysis revealed that microbes related to vaginal dysbiosis were also transcriptionally active, demonstrating the significant advantage of RNA-based methodologies. Although the endometrial microbiome, which is primarily studied via DNA sequences, has been suggested as a prognostic biomarker for female reproductive health, future studies should more clearly differentiate between the microbiome and microbiota. However, as RNA-based technologies for low microbial biomass are still being developed, larger studies are needed to confirm and support the findings of this study.

## Supplementary Material

hoag001_Supplementary_Data

## Data Availability

The data presented in the study are deposited in the NCBI SRA Database, accession number PRJNA1247240.
