# Supplementary material for: Comprehensive 16S rRNA gene sequencing and meta-transcriptomic analyses of the female reproductive tract microbiota: two molecular profiles with different messages
Source: Hum Reprod Open. 2026 Jan 6;2026(1):hoag001. doi: 10.1093/hropen/hoag001 (PMC12866996; doi:10.1093/hropen/hoag001)
Supplement: hoag001_Supplementary_Data [file hoag001_supplementary_data.zip › Supplementary Figures.docx]

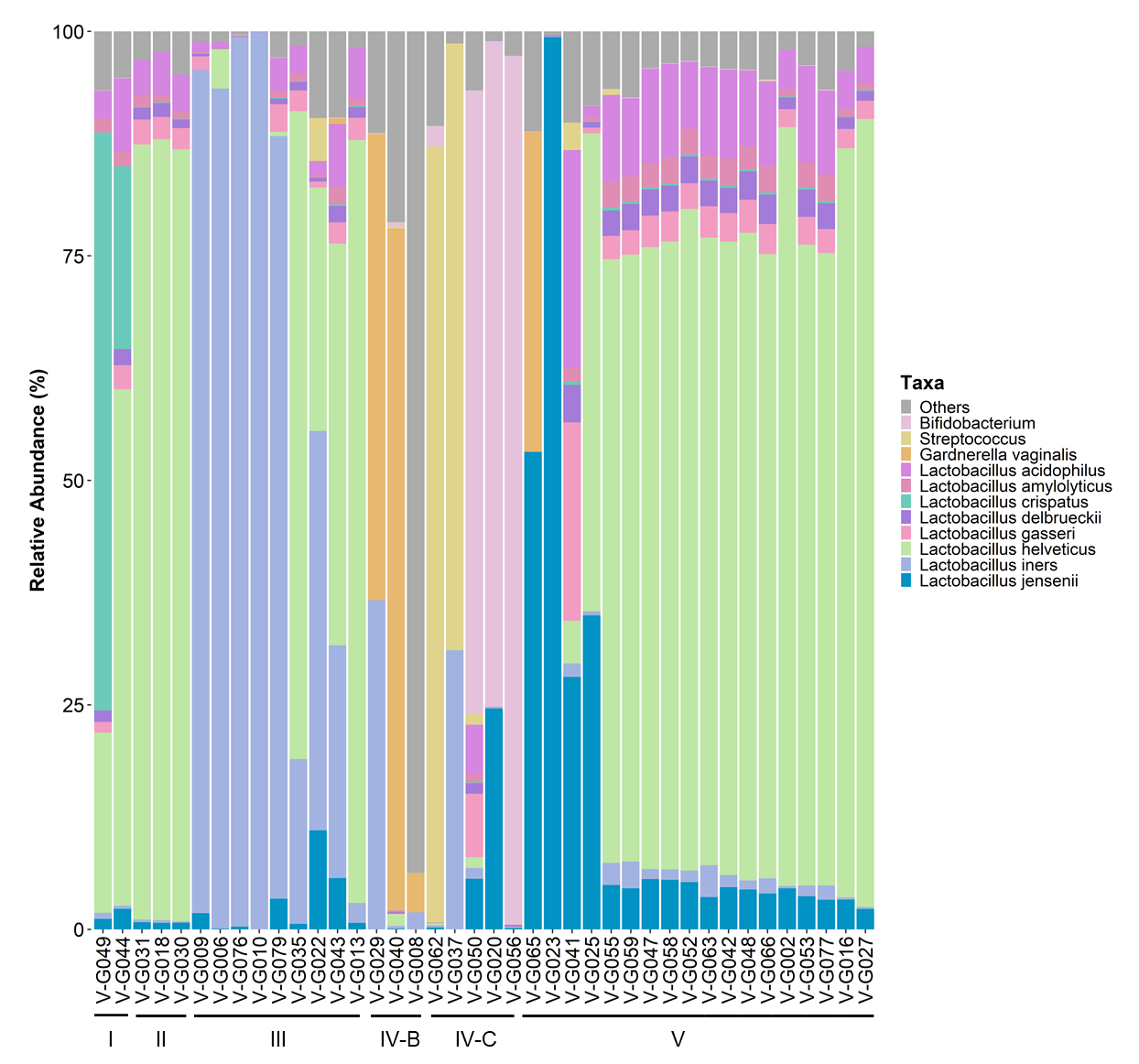


**Supplementary Figure S1. Community state types (CSTs) of vaginal samples analysed using 16S rRNA gene sequencing in the general cohort.** The microbial composition is plotted after decontamination. Bacteria with relative abundance less than 1% were grouped as “Others”.


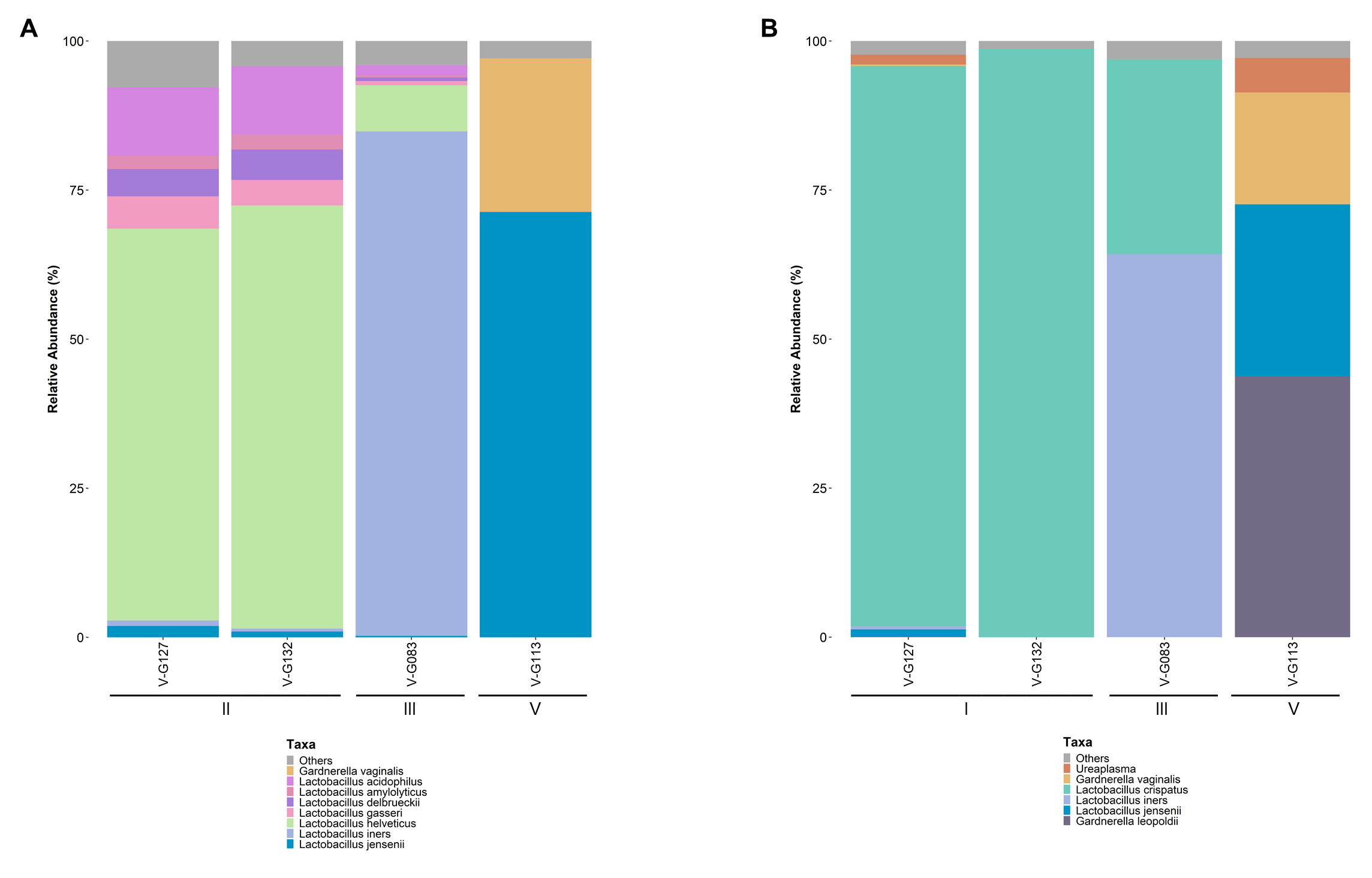


**Supplementary Figure S2.** Community state types (CSTs) of vaginal samples analysed using 16S rRNA gene sequencing (A) and meta-transcriptomics (B) in the validation cohort. The microbial composition is plotted after decontamination. Bacteria with relative abundance less than 1% were grouped as “Others”.


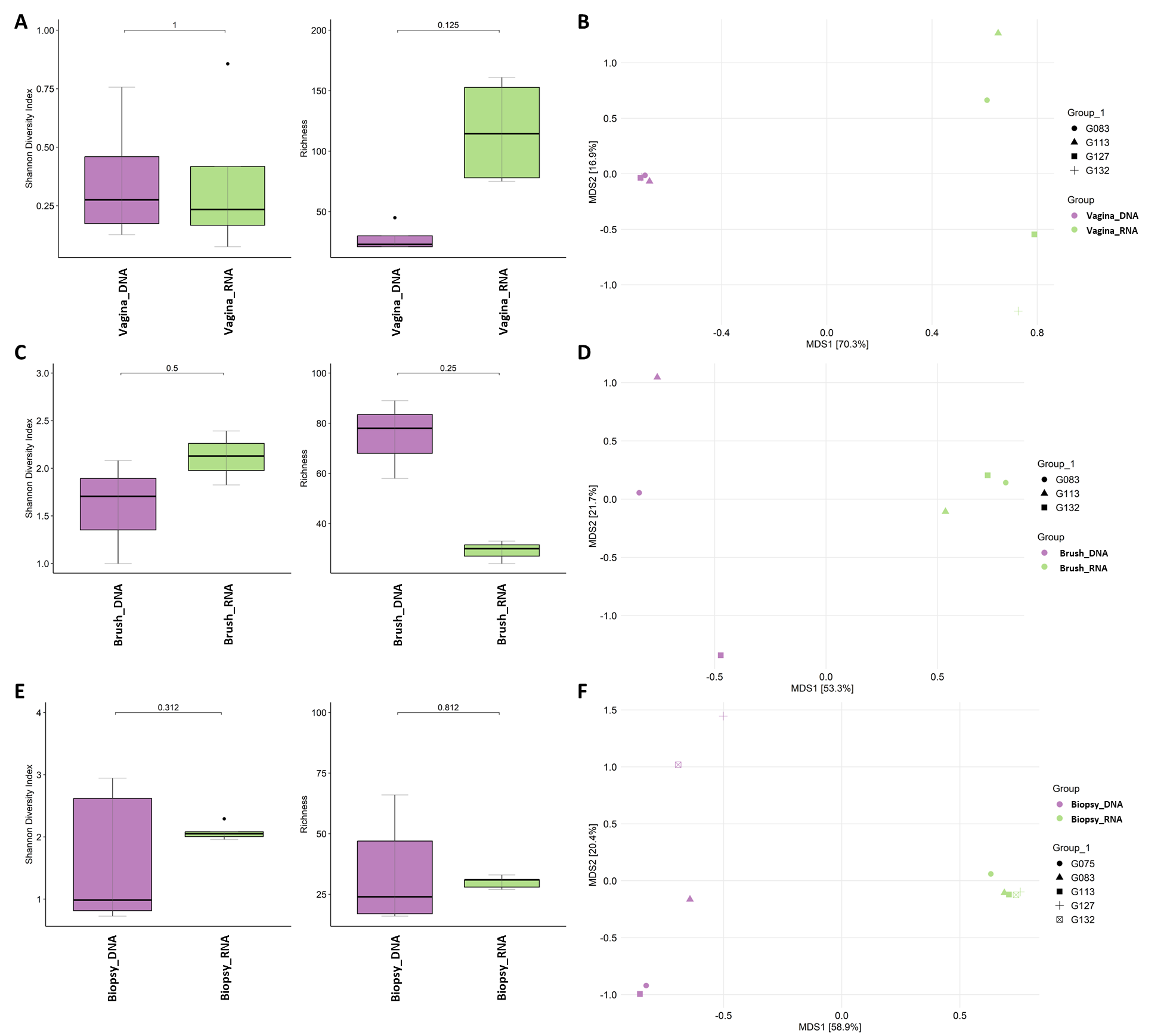


**Supplementary Figure S3.** Microbial alpha and beta-diversity measures in vaginal, endometrial brush and endometrial biopsies (DNA and RNA) samples in the validation cohort. (A, C, E) Shannon index and observed richness. (B, D, F) Principal coordinates analysis (PCoA) based on the Bray–Curtis dissimilarity (Adonis PERMANOVA, all R^2^ > 0.5, all p-values > 0.05).
